# Genetic outline of the hermeneutics of the diseases connection phenomenon in human

**DOI:** 10.18699/VJGB-23-03

**Published:** 2023-03

**Authors:** E.Yu. Bragina, V.P. Puzyrev

**Affiliations:** Research Institute of Medical Genetics, Tomsk National Research Medical Center of the Russian Academy of Sciences, Tomsk, Russia; Research Institute of Medical Genetics, Tomsk National Research Medical Center of the Russian Academy of Sciences, Tomsk, Russia Siberian State Medical University, Tomsk, Russia

**Keywords:** diseases connection phenomenon, syntropy, dystropy, comorbidity, hermeneutics, феномен сочетания болезней, синтропия, дистропия, коморбидность, герменевтика

## Abstract

The structure of diseases in humans is heterogeneous, which is manifested by various combinations of diseases, including comorbidities associated with a common pathogenetic mechanism, as well as diseases that rarely manifest together. Recently, there has been a growing interest in studying the patterns of development of not individual diseases, but entire families associated with common pathogenetic mechanisms and common genes involved in their development. Studies of this problem make it possible to isolate an essential genetic component that controls the formation of disease conglomerates in a complex way through functionally interacting modules of individual genes in gene networks. An analytical review of studies on the problems of various aspects of the combination of diseases is the purpose of this study. The review uses the metaphor of a hermeneutic circle to understand the structure of regular relationships between diseases, and provides a conceptual framework related to the study of multiple diseases in an individual. The existing terminology is considered in relation to them, including multimorbidity, polypathies, comorbidity, conglomerates, families, “second diseases”, syntropy and others. Here we summarize the key results that are extremely useful, primarily for describing the genetic architecture of diseases of a multifactorial nature. Summaries of the research problem of the disease connection phenomenon allow us to approach the systematization and natural classification of diseases. From practical healthcare perspective, the description of the disease connection phenomenon is crucial for expanding the clinician’s interpretive horizon and moving beyond narrow, disease-specific therapeutic decisions.

## Abstract (ru)

Структура заболеваний у человека неоднородна, характеризуется различными вариантами сочетаний болезней, включая сопутствующие патологии, связанные общим патогенетическим механизмом, а также болезни, редко проявляющиеся совместно на фенотипическом уровне. В последнее время отмечается рост интереса к изучению закономерностей развития не отдельных болезней, а целых семейств, связанных общими патогенетическими механизмами и общими генами, вовлеченными в их развитие. В результате установлен существенный генетический компонент, контролирующий образование конгломератов болезней сложным образом, через функционально взаимодействующие модули отдельных генов в генных сетях. Аналитический обзор исследований по проблематике разных аспектов сочетания болезней и является целью настоящей работы. В обзоре использована метафора герменевтического круга для познания структуры закономерных связей между болезнями, приведены концептуальные рамки, связанные с множественностью заболеваний у индивида. Рассмотрена существующая терминология применительно к ним, среди которых мультиморбидность, полипатии, коморбидность, конгломераты, семейства, «вторые болезни», синтропия и другие. Приведены ключевые результаты, чрезвычайно полезные, прежде всего, для описания генетической архитектуры болезней многофакторной природы. Обобщения по проблеме исследования феномена сочетания болезней позволяют приблизиться к систематизации и естественной классификации болезней. С точки зрения практического здравоохранения описание феномена сочетания болезней имеет решающее значение для расширения интерпретационного горизонта клинициста и выхода за пределы узких, ориентированных на конкретную болезнь терапевтических решений.

## Introduction

We live in the “Many Worlds in One” (Vilenkin, 2010) and this One World amazes us with the mystery and the universality of the connections of phenomena, the variety of evolutionary and historical events. These events take place both on a cosmic scale and on a planetary scale and the Earthlings (humanity) are the same universality of connections between themselves and the surrounding world. These connections are formed naturally or randomly, they have a long phylogenetic history of 4 billion years and only a hundred-year ontogenetic history of each individual. The structure of “human” connections, which appears in metabolic and morphophysiological variability, forms the basis of medical assessments – the norm or the disease. Since the beginning of the century, a new approach to the study of these issues – the network analysis – has emerged in biology and medicine. The network analysis is an attempt to understand the laws governing all kinds of networks, from the social to the complex gene networks that rule over all cells and traits, determining health or disease (Barabási et al., 2011).

The human genome, as the assemblage of all genes of the Homo sapiens species, is in a complex and not fully understood relationship with the environment and society. The peculiarity of such a relationship between genome and phenome is a difference often noted now: the genome is limited (approximately 3 billion base pairs in humans), the phenome is not limited (its limit depends on how far we want to go) (Paigen, Eppig, 2000). A century before the “genomic revolution” took place, in the 1930s, the outstanding Russian geneticist Alexander S. Serebrovskiy, discussing the problem of organic evolution, defined this problem as an “infinite-finite contradiction” in the “unity of an infinite number of traits and a finite number of genes” (Serebrovskiy, 1973).

In such an infinite world with an infinite number of traits, it is always possible (although it is not easy) to observe and identify traits connected to each other, including those related to pathology. In the clinic, this phenomenon forms the basis for diagnosis and healing, and stable combinations of certain disease traits represent an independent subject of research – the phenomenon of connection of diseases or the diseases connection phenomenon (DCP).

In 1970, the American physician and specialist in the field of epidemiology of non-communicable diseases, Alvan R. Feinstein, proposed the term “comorbidity” for combinations of diseases in individuals. Comorbidity means the manifestation of an additional clinical condition that exists or occurs in addition to the index disease under consideration (Feinstein, 1970). Such a clinical condition may be a disease, a pathological syndrome, pregnancy, a long-term “strict” diet, or a complication of drug therapy. Comorbidity is a complex of several diseases (megaforms, conglomerates) that exist simultaneously in individual patients and are observed much more frequently than would be expected in a random distribution.

The popularity of the term “comorbidity” is striking, especially among clinicians: there is the International Research Community on Multimorbidity (IRCMo), the Journal of Multimorbidity and Comorbidity (https://journals.sagepub.com/ description/COB) has been published since 2010, and there is an online medical platform for discussing the diagnosis and treatment of patients with comorbid diagnoses (https:// nexusacademy.ru/about). The author of the term “comorbidity” is credited with the discovery that “clarified” the interpretation of comorbid pathology (Vertkin, 2015). And yet, there is still a feeling of overestimation of “clarity” in the understanding of the phenomenon and the term. It is similar to the situation described in the novel of the famous Nobel laureate William Faulkner: “They all talked at once, their voices insistent and contradictory and impatient, making of unreality a possibility, then a probability, then an incontrovertible fact, as people will when their desires become words”(William Faulkner. The Sound and the Fury. 1929.)

And yet, we must agree that the term “comorbidity” has proved especially successful for clinicians. It became an umbrella term for numerous names of combinations of diseases, variants of two or more forms of pathology in patients and, often, in their closest relatives. Sometimes such diseases are called background or concomitant diseases. In general, according to our calculations, the pool of names for such combinations of diseases includes more than 30 terms. Among them: multimorbidity, polypathies, comorbidity, conglomerates, families, “second diseases” and others. Most often there are diseases that have a “common root” (related pathogenesis, trans-syndromal comorbidity), although other combinations of diseases show nothing in common in pathogenesis (transnosological comorbidity). Note that specific terminological studies are limited, and as a result we see no consensus (Azaïs et al., 2016; Navickas et al., 2016). However, in the current situation the object of the study is defined, it is “comorbid patient” (Vertkin, 2015). Good quality clinical and epide-miological data is accumulated, which came in time to become the basis for implementation of “omics” approaches to research on the DCP problem. And there is very serious content and a rather serious genetic aspect. This is the subject of this article.

## Conceptual toolkit in the genetic study of the DCP

Here we present a set (assemblage) of views (principles, concepts) connected with each other and forming a unified system, that is useful, in our opinion, for understanding (interpretation, explanation) of the DCP. Let us use a metaphor – the “hermeneutic circle” – which describes the mutual agreement between the individual (part) and the whole, like a hermeneutical rule: we must understand the whole in terms of the detail, and the detail in terms of the whole (Gadamer, 2010). If we consider the DCP as a “whole”, it would be reasonable to include as “the details” (the components of the hermeneutic circle) the fragments of concepts (doctrines, principles) of outstanding clinical geneticists, such as the Soviet neurologist Sergey N. Davidenkov (1880–1961), the American geneticist Victor A. McKusick (1921–2008), the German pediatrician Meinhard von Pfaundler (1872–1947) and the now living German-American clinician John M. Opitz. All of them are, at the same time, geneticists and, most importantly, practicing physicians who investigated the polymorphism of disease manifestation and the mysterious phenomenon of a combination of several pathologies in one patient.

Without keeping the chronological order of their publications, we follow the intended logic in presenting the structure of the hermeneutic circle, i. e., those “details” that can be useful in interpreting the DCP as a “whole”.

“Lumpers” and “splitters” (McKusick, 1969). In the 1960s, a discussion was opened in the medical genetics community – what is the “nosology” of genetic diseases? Mainly Mendelian diseases were discussed, but also diseases with an inherited predisposition (multifactorial diseases, MFDs). Phenotypically, patients represent a huge clinical diversity, and possibilities of clarifying the etiology of diseases by molecular genetic or cytogenetic methods were limited in those years. So, physicians-researchers were quite free to classify the patients by combining or separating them. However, during the discussion of this problem, an important generalization was proposed and it was the principles of medical genetics: pleiotropism, variability (polymorphism) and genetic heterogeneity (McKusick, 1968). These principles, above all, can be considered stabilizing the semantic context of understanding the DCP. Today’s systematists of human pathology also rely on these principles (Biesecker, 1998; Brunner, van Driel, 2004). Moreover, with the advances in genomic medicine, it became possible to describe the genetic architecture of multifactorial diseases, which is understood as the number of genetic polymorphisms that affect the risk of disease, the distribution of their allelic frequencies and their effect sizes, as well as their genetic mode of action (additive, dominant and/or epistatic, pleiotropic) (Wray et al., 2008).

Syndrome as pleiotropy, conditional tropism hypothesis (Davidenkov, 1947; Opitz, Neri, 2013). The word “syndrome” was first used in English in 1541, as noted by (Opitz, Neri, 2013), and is still used to indicate a common cause rather than simply a set of symptoms. The same authors also evaluate another dictionary definition – the syndrome, as a concurrence of manifestations “characterizing a specific disease”, a greater-than-chance concurrence of identical or very similar sets of manifestations in two or more individuals suggesting similar pathogenesis, subject to causal verification through the discovery of physical, infectious, toxicological, or genetic factors (Opitz, Neri, 2013).

Today, biochemical and refined molecular/cytogenetic methods identify genetic causes, epigenetic modifications in combined phenotypes or syndromes with high accuracy. The explanation of such combinations, their persistence or “dividing” in descendants, the severity of manifestations of similar combinations, as well as the interpretation of the relationship between multiple variations of the norm or minor anomalies with their advanced forms of pathology was suggested by Sergey N. Davidenkov in the conditional tropism hypothesis (1947). He used the evolutionary-genetic approach to analyze more than one hundred nosological forms of human nervous diseases. The frequency of combined appearance of the diseases of the nervous system in one patient or in one family is explained by conditional tropism: in addition to its own influence on the nervous system development, the pathological property (gene) also has the ability to dramatically enhance the phenotypic expression of other genotype features “moving into the same direction” and including numerous variants. So, for example, a mild excavation of the foot can take the form of a severe Friedreich’s deformity.

Associations, syntropies and dystropies, the transitive association hypothesis (Pfaundler, Seht, 1921; Blair et al., 2013). The renowned textbook for the diagnosis of congenital diseases (Jones, 2011) defines associations as combinations of congenital anomalies that have no well-defined etiology and occur together more often than expected by chance alone. Since its inception, the concept of “associations” has engendered feelings of unease and vagueness, as noted (Opitz, Neri, 2013). They agreed on two variants in the definition of the term: coincidental concurrence (simple rencontre or simple juxtaposition) and combination of anomalies (close connection, polytopic defect of a body area). In the 1900s, new designations of essentially the same associations appeared: but the term “multiple abarts” (from the German abart, malformation) was proposed for hereditary diseases and congenital malformations, and “syntropy” (Syntropie in German) (Pfaundler, Seht, 1921) was proposed for common multifactorial diseases occurring in one patient at the same time. They not only termed the “mutual disposition, attraction” of the two diseases by the term “syntropy”; in addition, on the basis of abundant clinical data and tens of thousands autopsies Pfaundler and Seht recorded another pathological condition opposite to syntropy – “mutual repulsion”, incompatibility (incongruity, dissociation) and named it “dystropy” (Dystropie in German). At the same time, intermediate, to a certain extent random and “neutral states” also got their name, “neutropy” (Neutrotropie in German). According to these researchers, the term “syndrome” can also be regarded as syntropy, because it means a “selective relationship” of its constituent traits. Another property of the unity of pathological conditions is the appearance of at least two diseases in one patient at the same time (synchrony). Thus, syntropy, syndrome, synchrony (“3S”) are related concepts and the main factor uniting them is a similar pathogenesis. For example, in relation to atherosclerosis, diabetes and obesity is a “common root” (Stein O., Stein Y., 1995).

In our current definition, syntropy is a natural-species phenomenon of a combination of two or more pathological conditions (nosologies or syndromes) in an individual and his closest relatives, non-random and having an evolutionary genetic basis; it is a part (an extract) of the human phenome, comprised of a landscape of interacting traits and diseases, reflecting continual molecular-genetic causality (Puzyryov, 2002; Puzyrev et al., 2010). The genes involved in the development of syntropies are called syntropic genes. More precisely, syntropic genes are a set of functionally interacting genes localized throughout the genome, coregulated and involved in a metabolic pathway common to a given syntropy (Puzyryov, 2002; Puzyrev et al., 2010). In the case when regulatory relationships lead to the mutual exclusion of certain phenotypes at the clinical level (dystropy), such genes are termed dystropic in relation to the relevant phenotypes. There is some semantic similarity of the concepts of “syntropic and dystropic genes” with the term “core genes”, which were discussed in the recently proposed omnigenic model of complex disease (Boyle et al., 2017).

Finally, let us talk about the transitive genetic association hypothesis. The transitive associations are another form of association from the described above, syntropy (association in the conventional sense and the most common form) and dystropy (dissociation). David R. Blair et al. (2013) hypothesized that statistically significant comorbidities between complex (MFDs) and Mendelian diseases represent a type of genetic association, in which a non-Mendelian phenotype is mapped to the genetic loci that cause the Mendelian disease. In fact, transitive associations are a kind of syntropy, but the phenotype is the result of a combination of complex and Mendelian disease. According to the authors of the hypothesis, such conditions represent about half (54 %) of all comorbid diseases (Blair et al., 2013).

Classification of variants of diseases connection in humans. There is no generally accepted classification of the DCP. Moreover, the tasks of systematization, understanding of the general properties that fix regular connections, in all the variety of such combinations, have not been formulated; the existing attempts to classify such pathological phenomena are still fragmented and conditional. Most often, they are descriptive in nature. This is especially true for the clinical classification of connections designated by the term “comorbidity”, and carriers of such pathological features are referred to as “comorbid patients” (Vertkin et al., 2012). Now we can also confirm the attempts to systematize the concept of “syntropy” (Krylov, 2000): by the mechanisms of formation (etiological, pathogenetic, age-related, iatrogenic, random), by the time of occurrence (congenital, delayed, simultaneous, successive) and by clinical significance (inert, interference).

**Fig. 1.**
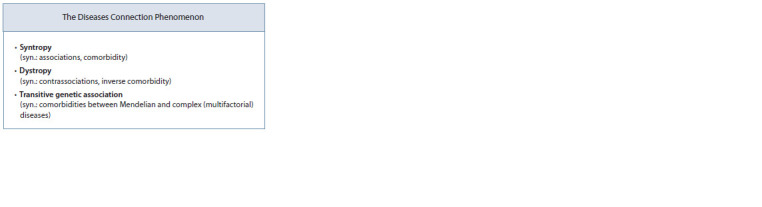
Classification of disease connection forms in humans.

Previously, we (Puzyrev, 2015) proposed the identification of the following forms of diseases connection in individual patients (Fig. 1). The proposed systematization of the DCP forms is also descriptive, but the elements of intrinsic classifications can also be seen in it. This is associated, among other things, with the designation of the key terms of connection characteristics: association and syntropy. There are several subject areas in scientific research (besides medicine), in which the term “syntropy” is used. Viktor B. Vyatkin (2016) designates three fields of science in which the concept of “syntropy” takes an important place, proposing a classification of syntropy (in order of the beginning of their use) into: medical (Pfaundler – von Seht syntropy), biophysical (Fantappiè – Szent-Györgyi – Fuller syntropy), informational (Vyatkin syntropy). In our opinion, these two additional types of syntropy not only have an independent significance, but are also important for the essential understanding of biological processes, including both in general pathology and in the particular pathogenesis of the DCP.

Note that the multiplicity of diseases in an individual is a long-standing problem that had attracted the attention of researchers before the widespread use of the “comorbidity” term. The commonality of the mechanisms of development of nonrandom pathological connections is reflected in the names of relevant concepts: “the sum of homeostasis diseases” (Dilman, 1968), “diseases of adaptation” (Kaznacheev, 1980), “cardiovascular disease continuum” (Dzau et al., 2006), “metabolic syndrome” (Reaven, 1988). It is important to consider this problem from the genetic perspective, the concepts of diseasome (Goh et al., 2007) and network medicine (Barabási et al., 2011; Kolchanov et al., 2013).

Generalizations on the problem of studying the DCP allow us to approach the intrinsic classifications of the phenomenon. It is important. As Mikhail D. Golubovsky (2006) noted, a good system is an event in science, a conceptual discovery, a new vision of harmony in the chaos of facts. That is why the inclusion of classifications in the hermeneutic circle seems useful.

## Actual data on the DCP study

Syntropies (comorbidity)

Syntropy is widespread and more common than we imagine. For example, the 438 common diseases registered in the UK Biobank patient histories (https://www.ukbiobank.ac.uk/) form more than 11,000 possible combinations (Dong et al., 2021). The global nature of the problem has initiated a huge number of studies, mainly of an epidemiological kind. In 2021 alone, the query ‘comorbidity’ found 34,185 medical and biological articles in the US National Center for Biotechnology Information database (https://pubmed.ncbi.nlm.nih.gov/). Currently, more than 50 million people aged 65 and older – nearly half of Europe’s population – have two or more diseases at the same time (Rijken et al., 2018). The number of comorbid patients is predicted to continually increase, affecting up to 68 % of the population by 2035 (Kingston et al., 2018).

Molecular causes of phenotypic connections remain largely unknown, despite active research in this field (Reynolds et al., 2021; Jia et al., 2022; Quick et al., 2022; Shnayder et al., 2022; Wang et al., 2022). Through these studies, it became evident that a significant proportion (46 %) of comorbid conditions is caused by a common component at the level of genes, SNPs, and gene networks interactions (Dong et al., 2021), that in general reflects their pathogenetic relationship. For example, the HLA-DQB1, TLR1, WDR36, LRRC32, IL1RL1, GSDMA, TSLP, IL33, SMAD3 genes involved in the pathogenesis of certain allergic diseases are critical for the development of phenotype according to the “atopic march” scenario (Ferreira et al., 2014). Meanwhile, in terms of pathogenesis, seemingly non-obvious connections between diseases are revealed. The existence of many of these connections was not previously even assumed. Varicose veins disease, according to genetic correlations analysis, is associated with fluid intelligence, prospective memory and educational attainment (Shadrina et al., 2019), and autism is positively correlated with allergic rhinitis and autoimmune disorders (Rzhetsky et al., 2007). A significant addition to the identification of common genes for comorbid conditions is the study of the biological processes in which these genes are involved (Rubio-Perez et al., 2017). The use of such approaches provides a more complete picture of the connections of diseases and common pathogenetic pathways. Knowledge of these connections can be widely applied, including treatment of comorbid patients.

**Fig. 2.**
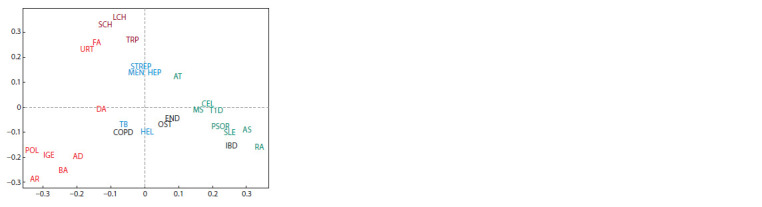
The results of multidimensional scaling of multifactorial diseases based on the commonality of the genes associated with them (adapted from Freydin et al., 2015). Abbreviations for diseases: AD – atopic dermatitis, AR – allergic rhinitis, AS – ankylosing spondylitis, AT – autoimmune thyroiditis, BA – bronchial asthma, CEL – celiac disease, COPD – chronic obstructive pulmonary disease, DA – drug allergy, END – endometriosis, FA – food allergy, HEL – Helicobacter infection, HEP – viral hepatitis, IBD – inflammatory bowel disease, IGE – immunoglobulin E level, LCH – leishmaniasis, MEN – meningococcal infection, MS – multiple sclerosis, OST – osteoporosis, POL – pollinosis, PSOR – psoriasis, RA – rheumatoid arthritis, SCH – schistosomiasis, SLE – systemic lupus erythematosus, STREP – streptococcal infection, T1D – type 1 diabetes mellitus, TB – tuberculosis, TRP – trypanosomiasis, URT – urticaria.

Based on our own research findings on the genetic component of allergic diseases (Freidin et al., 2015) on the one hand, we established the molecular connection of most allergic diseases. On the other hand, with regard to the molecular relationships of allergic diseases with other diseases, we noted their proximity to infectious diseases and a marked distance from autoimmune diseases (Fig. 2).

The TLR4, CAT, ANG/RNASE4 genes can make the greatest contribution to the comorbidity of bronchial asthma and hypertension, indicating the importance of inflammation, neovascularization and oxidative stress for the pathogenesis of both diseases (Bragina et al., 2018). The development of bronchial asthma phenotypes in combination with cardiovascular/ metabolic disorders is associated with certain genetic variants that affect gene expression, including CAT, TLR4, ELF5, ABTB2, UTP25, TRAF3IP3, NFKB1, LOC105377347, C1orf74, IRF6 and others, in the target organs of the studied disease profile (Bragina et al., 2022).

Syntropic genes are involved in pathogenesis through complex interactions with other genes, proteins, and environmental factors, which collectively affect the clinical manifestations of comorbidities. In most cases, abnormalities in syntropic genes are localized mainly in non-coding RNAs and intergenic regions functionally associated with the regulation of gene transcription (Dong et al., 2021). In turn, the transcription of syntropic genes depends on epigenetic mechanisms, in particular DNA methylation (Ferreira et al., 2017), which indicates a modifying role of environmental influences on complex phenotype development.

Many syntropic genes are known drug targets for therapy, in particular allergic (FLG, IL13, IL1RL1, IL6R, INPP5D, NDFIP1, PTGER4, TSLP, STAT6) (Ferreira et al., 2017), bronchopulmonary and cardiovascular (EDNRA, ADRB1, ADRB2) diseases (Zolotareva et al., 2019; Dong et al., 2021). More than eight thousand drugs target genes involved in the development of comorbid conditions (Dong et al., 2021). Theoretically, such results not only highlight the important contribution of genes to phenotypic correlations, but also provide an opportunity for drug repurposing to target common genetic components of syntropic diseases.

Dystropies (“diametrical diseases”)

The contrast for syntropy is the diseases that manifest by the phenotypic conflict of one pathological condition with another (dystropy). Dystropy affects various diseases including immune, oncological, neurodegenerative, cardiovascular, autoimmune and others. The spectrum of molecular mechanisms underlying this phenomenon also seems to be very diverse. Research on dystropy focuses on the search for molecular and genetic differences between the diseases. As a result of these studies, differences in the transcription of the same genes in different diseases have been established. Using the example of oncological and neurodegenerative diseases dystropy (Catalá-López et al., 2014), it was revealed that differentially expressed genes are mainly associated with DNA repair, mitochondrial function, stabilization of p53, regulation of angiogenesis, cell cycle, metal ion transport, glucose transport, regulation of apoptotic processes, myeloid leukocyte activation and phagocytosis, mTORC1 and KRAS signaling (Forés-Martos et al., 2021; Pepe et al., 2021). Transcriptional changes in oncogenesis are highly variable; some genes may be activated in some forms of cancer, but suppressed in others, which is probably associated with the features of complex genetic and epigenetic disorders (Zhao et al., 2016). At the same time, common patterns are recorded. In particular, Ibáñez et al. (2014) identified the genes MT2A, MT1X, NFKBIA, AC009469.1, DHRS3, CDKN1A, TNFRSF1A, CRYBG3, IL4R, MT1M, FAM107A, ITPKC, MID1, IL11RA, AHNAK, KAT2B, BCL2, PTH1R, NFASC that are simultaneously activated in several disorders of the central nervous system (Alzheimer’s disease, Parkinson’s disease, schizophrenia) but are suppressed in oncological diseases.

The examples above indicate that phenotypic suppression is mediated by genetic factors. In some cases, potentially “harmful” alleles can be beneficial, creating some kind of trade-off between an increased risk of developing certain diseases and a low risk of developing others. Trade-offs are inevitable, because the complex integrated functioning of the whole organism needs several interacting parts to work together to perform certain functions. Such integration can lead to a dilemma often called the “cost of complexity” (Wagner et al., 2008), resulting from multiple interacting parts working together to successfully perform a function. Alteration of any part will inevitably negatively affect other features, altering function and reducing overall performance or fitness. Thus, the mechanistic basis for the trade-offs may be focused on pleiotropic genes involved in the biological pathways shared between different traits (Mauro, Ghalambor, 2020). In accordance with this suggestion, the observed divergent nature of the transcription of some genes thought to be important for dystropy can be expected. Diametrical disorders have the intrinsically bidirectional nature of biological processes, whereby expression or activation of genes can be increased or decreased from some optimal value (Crespi, Go, 2015).

Dystropy is significantly formed by drug therapy, because drugs can be connected with the regulation of common molecular processes of phenotypically polar diseases. For example, the use of anticholinesterase agent galantamine and the selective monoamine oxidase inhibitor selegiline in neurodegenerative diseases has anticancer effects (Lazarevic-Pasti et al., 2017; Ryu et al., 2018). Two drugs for breast cancer therapy (exemestane and estradiol) reduce the risk of Alzheimer’s disease and other dementias (Branigan et al., 2020; Guglielmotto et al., 2020).

Transitive genetic associations

Genes that can harbor mutations underlying rare and highly penetrant Mendelian diseases affect the development of more common forms of diseases. The effect of mutations can be either a predisposing factor for disease development or vice versa, a suppressor of phenotype manifestations. There are various estimates of the involvement of Mendelian genes in the phenotypic expansion of multifactorial pathology. About 300 genes associated with common diseases in genome-wide studies underlie a number of Mendelian diseases (Lupski et al., 2011). By some estimates, the proportion of Mendelian genes in the structure of multifactorial diseases is approximately 23 % (Spataro et al., 2017), but with the growth of genomewide sequencing data, this amount is likely to increase significantly. In terms of specific pathology, 11 (ABCG8, LCAT, APOB, APOE, LDLR, PCSK9, CETP, LPL, LIPC, APOA5 and ABCA1) out of 30 genes associated with serum lipoprotein concentrations are involved in monogenic disorders of lipid metabolism (Kathiresan et al., 2009). These genes, which are causative variants of both Mendelian disorders and the risk of multifactorial diseases, tend to have higher functional significance and higher expression levels than genes only associated with common diseases. Furthermore, genetic variants in conditionally “Mendelian” genes tend to present higher odds ratios than variants on genes with no link to Mendelian disorders (Spataro et al., 2017).

The idea of a mutational burden materialization in common pathology is not new. The experimental basis for this phenomenon was the publication of Michael S. Brown and Joseph L. Goldstein (Brown, Goldstein, 1986), which showed that patients with heterozygous mutations in the low-density lipoprotein receptor (LDLR) gene, along with familial hypercholesterolemia, have coronary atherosclerosis and myocardial infarction. In 2013, David R. Blair (Blair et al., 2013) formulated a hypothesis about the transitivity of rare Mendelian variants into a pathological “allelic continuum” in a wide range of final phenotypic effects from monogenic to complex multifactorial diseases. To date, extensive factual material has been accumulated to support this hypothesis. Carriers of FLG gene mutations associated with loss of filaggrin function have an increased risk of developing atopic dermatitis (Sandilands et al., 2007) and bronchial asthma in the context of atopic dermatitis, while at the same time the risk of asthma without atopic dermatitis is reduced (Palmer et al., 2006). This finding suggests that FLG gene mutations are an important risk factor for atopy in general, but with different chances for a particular phenotype. Carriers of Gaucher disease mutations, mainly L444P and N370S in the glucocerebrosidase (GBA) gene, have an increased risk of Parkinson’s disease (Sidransky et al., 2009). Heterozygous carriers of mutations in the cystic fibrosis transmembrane regulator (CFTR) gene are predisposed to idiopathic chronic pancreatitis (Weiss et al., 2005) and chronic obstructive pulmonary disease (Divac et al., 2004).

Various approaches are used to gain knowledge about the active contribution of Mendelian disease genes as causative genes for multifactorial diseases. For example, based on the prioritization of data from genome-wide associative studies of various forms of cardiomyopathies, it was found that 70 % of the hypertrophic and 56 % of the dilated cardiomyopathy genes are associated with various Mendelian diseases. This finding suggests that the existing dichotomous classification of diseases – monogenic and multifactorial – has become irrelevant and requires rethinking taking into account new knowledge about the genetic structure of susceptibility (Nazarenko et al., 2022).

The potential of separate gene mutations is evaluated as protective factors in relation to oncological diseases. In particular, activation of apoptosis and autophagy by mutant huntingtin (Gomboeva et al., 2020), as well as the oncotoxic function of CAG repeats (Murmann et al., 2018), the expansion of which causes the Huntington’s disease, may prevent the development of most types of cancer in patients with this hereditary disease (Catalá-López et al., 2014). The molecular oncoprotective mechanism of the Laron dwarfism mutation (OMIM #262500) (NM_000163.5(GHR):c.594A>G (p.Glu198=)) in the growth hormone receptor gene is mediated by effects on the activity of genes involved in the control of the cell cycle, mobility, growth and oncogenic transformation (Werner et al., 2020).

Loss of function of individual proteins due to loss-offunction mutations provides specific resistance against some common phenotypes. Protection against type 2 diabetes is associated with carrying a mutation in the zinc transporter type 8 gene (SLC30A8) that leads to the synthesis of a truncated protein (Flannick et al., 2014). As a consequence of the resulting deficiency of SLC30A8 gene function through the mechanism of haploinsufficiency, carriers of mutant alleles have better insulin secretion due to increased glucose sensitivity and proinsulin conversion in the pancreatic beta cells. Another example relates to nonsense mutations (Y142X, C679X, and R46L) in the proprotein convertase subtilisinkexin type 9 (PCSK9) gene underlying familial hypercholesterolemia (OMIM #603776); these mutations result in lower low-density lipoprotein cholesterol level (Cohen et al., 2005). Heterozygous carriers of F508del in the cystic fibrosis transmembrane regulator (CFTR) gene, which causes cystic fibrosis, are more resistant to infectious diseases such as cholera, typhoid fever and tuberculosis. Therefore, some authors attribute the high prevalence of cystic fibrosis in the modern human population to the adaptive advantage of mutation carriers (Bosch et al., 2017).

**Fig. 3.**
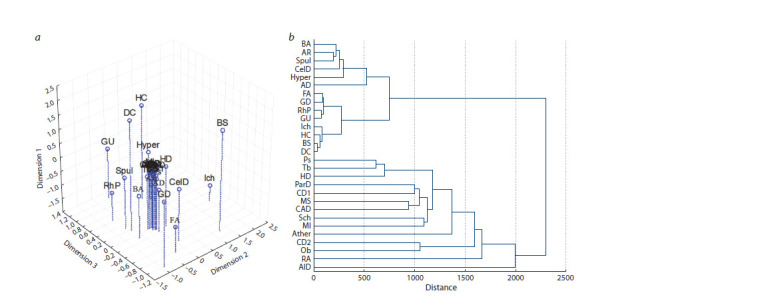
Modeling of relationships between multifactorial/monogenic diseases by commonality of associated genes, based on: multivariate scaling (a) and hierarchical cluster analysis (b). Abbreviations for diseases: AD – eczema (atopic dermatitis), AlD – Alzheimer’s disease, AR – allergic rhinitis, Ather – atherosclerosis, BA – atopic bronchial asthma, BS – Brugada syndrome, CAD – coronary artery disease, CD1 – type 1 diabetes, CD2 – type 2 diabetes, CelD – celiac disease, DC – dilated cardiomyopathy, FA – food allergy, GD – Gaucher’s disease, GU – gastric ulcer, HC – hypertrophic cardiomyopathy, HD – Huntington’s disease, Hyper – arterial hypertension, Ich – ichthyosis, MI – myocardial infarction, MS – multiple sclerosis, Ob – obesity, ParD – Parkinson’s disease, Ps – psoriasis, RA – rheumatoid arthritis, RhP – polyposis sinusitis, Sch – schizophrenia, Spul – pulmonary sarcoidosis, Tb – tuberculosis.

The results of the classification of some multifactorial and Mendelian diseases based on the genes associated with them have identified a large common genetic component of multifactorial diseases (as evidenced by their proximity to the center in Figure 3, a). Monogenic diseases are expectedly distant from them, with the exception of Huntington’s disease, which is not only close to other neurodegenerative diseases in the degree of gene commonality, but also has molecular similarities with infectious, autoimmune, and cardiometabolic diseases (see Fig. 3, b). Overall, in terms of the degree of genetic “commonality” and clustering, most of the diseases studied reflect the generally accepted classification of diseases. However, such modeling has a limitation, since it depends on the extent to which genes are studied, so we should expect a shift in the location of monogenic diseases. At present, the amount of genomic information is rapidly expanding, which brings us closer to filling the gap in the knowledge about disease-associated genes. But even after this gap is filled, a more difficult task remains: to understand the mechanisms of manifestation of the mutation effect and to map the genetic interactions of mutations in different genes, which are combined in a certain way due to structural and molecular interaction (Diss, Lehner, 2018), contributing to phenotypic diversity.

## Conclusion

The last decades have been an important milestone for genomic research due to the possibilities of high-throughput technology and the enormous amount of data obtained. It is expected that between 100 million and 2 billion human genomes could be sequenced by 2025, far exceeding growth in other dynamically developing fields that generate Big Data: astronomy, YouTube and Twitter (Stephens et al., 2015). The authors of the aforementioned paper compare genomic research to a “four-headed beast” based on four main demands in genomics throughout the life cycle of the datasets generated by sequencing – acquisition, storage, distribution, and analysis (Stephens et al., 2015). Of these four demands, the greatest effort is required to analyze and comprehend the results obtained, to unravel the complex relationship between genetic variants and phenotypes. This relationship is to a large extent a stochastic process, limited by the genome on the one hand and environmental factors on the other. Consequently, rational ways to comprehend biologically complex objects in the world of Big Data are still relevant.

The results of the study of the diseases connection phenomenon (comorbidity, syntropy/dystropy) accumulated in the scientific literature lead to the necessity and possibility of approaching such a vision of generalization, which was outlined by the outstanding Carl R. Woese in his paper: “...the essence of biology lies not in things as they are, but in things coming into existence” (Woese, Goldenfeld, 2009). In this context, our article attempts to consider the diseases connection phenomenon within the framework of the “hermeneutic circle” metaphor. It is important to note the historical continuity of scientific knowledge on the issue, which was originally based on a holistic view of the development of living organisms, ranging from ‘Geoffroyism’ (named after Étienne Geoffroy Saint-Hilaire), reflected in the principles of connexion, the unity of elementarity and integrity (Holodkovsky, 1915), to the manifestation of the complex tropism of hereditary factors (Davidenkov, 1947) and the principles of systematization in medical genetics (McKusick, 1968), and finally to the framework of modern concepts of network biology and medicine (Barabási et al., 2011; Kolchanov et al., 2013).

The progress of research on comorbidities has shown the insufficiently comprehensive nature of the existing terminology. For example, in contrast to the term “comorbidity”, which has become familiar in medical practice, the genetic discourse of the proximity of concomitant diseases is most fully interpreted by the terms “syntropy” and “dystropy”, reflecting the peculiarities of pathogenetic relationships between diseases. The pathogenetic principle of gene involvement in the development of comorbid diseases allowed to classify them as syntropic and dystropic genes (Puzyrev, 2015). Important in this context is the classification of genes on a mechanistic basis into nuclear/core and peripheral genes, that have omnigenic effects on the development of the pathological phenotype through trans- and cis-regulation (Boyle et al., 2017; Liu et al., 2019). It is obvious that, along with nuclear genes, peripheral genes are important objects for MFDs comorbidity studies, because their global activity in specific cell types determines cellular function and disease risk.

The molecular nature of comorbidities, which allows them to be connected in many, often non-fatal and even beneficial combinations, remains difficult to explain due to some “liberties of genome” determined by the dynamic and non-linear nature of the functioning of the system, regulated by feedbacks that can be disrupted in predictable but individual way. The degree of benefit or harm of such combinations of diseases of the conditional “adaptive phenotype” depends on the tradeoffs that are most obvious due to competition for the limited resources of the organism. Probably, vulnerability to some diseases with a relatively low risk of developing others is reduced to the establishment of some “price of complexity”, based on the pleiotropic action of genes.

Thus, the diseases connection phenomenon, described in clinical practice for a long time, is of independent interest for fundamental research. The DCP also becomes an additional way to elucidate the etiology and pathogenesis of complex diseases, in the study of which modern methodological and conceptual approaches are involved. On the other hand, the diseases connection phenomenon is important for practical healthcare, since its description is crucial for expanding the clinician’s interpretative horizon and moving beyond narrow, disease-specific therapeutic decisions. By expanding our knowledge of the molecular diversity of the human phenome, we can encourage the revision of current disease classifications (Piro, 2012), the identification in such classifications of subtypes with different prognosis for the patient and family members, individual responses to treatment (Manolio, 2013).

## Conflict of interest

The authors declare no conflict of interest.

## References

Azaïs B., Bowis J., Wismar M. Facing the challenge of multimorbidity.
J. Comorb. 2016;6(1):1-3. DOI 10.15256/joc.2016.6.71.

Barabási A.L., Gulbahce N., Loscalzo J. Network medicine: a networkbased
approach to human disease. Nat. Rev. Genet. 2011;12(1):56-
68. DOI 10.1038/nrg2918.

Biesecker L.G. Lumping and splitting: molecular biology in the genetics
clinic. Clin. Genet. 1998;53(1):3-7. DOI 10.1034/j.1399-0004.
1998.531530102.x.

Blair D.R., Lyttle C.S., Mortensen J.M., Bearden C.F., Jensen A.B.,
Khiabanian
H., Melamed R., Rabadan R., Bernstam E.V., Brunak
S.,
Jensen L.J., Nicolae D., Shah N.H., Grossman R.L., Cox N.J.,
White K.P., Rzhetsky A. A nondegenerate code of deleterious variants
in Mendelian loci contributes to complex disease risk. Cell.
2013;155(1):70-80. DOI 10.1016/j.cell.2013.08.030.

Bosch L., Bosch B., De Boeck K., Nawrot T., Meyts I., Vanneste D.,
Le Bourlegat C.A., Croda J., da Silva Filho L.V.R.F. Cystic fibrosis
carriership and tuberculosis: hints toward an evolutionary selective
advantage based on data from the Brazilian territory. BMC Infect.
Dis. 2017;17(1):340. DOI 10.1186/s12879-017-2448-z.

Boyle E.A., Li Y.I., Pritchard J.K. An expanded view of complex traits:
from polygenic to omnigenic. Cell. 2017;169(7):1177-1186. DOI
10.1016/j.cell.2017.05.038.

Bragina E.Y., Goncharova I.A., Garaeva A.F., Nemerov E.V., Babovskaya
A.A., Karpov A.B., Semenova Y.V., Zhalsanova I.Z., Gomboeva
D.E., Saik O.V., Zolotareva O.I., Ivanisenko V.A., Dosenko V.E.,
Hofestaedt R., Freidin M.B. Molecular relationships between bronchial
asthma and hypertension as comorbid diseases. J. Integr. Bioinform.
2018;15(4):20180052. DOI 10.1515/jib-2018-0052.

Bragina E.Yu., Goncharova I.A., Zhalsanova I.Z., Nemerov E.V., Nazarenko
M.S., Freidin M.B., Puzyrev V.P. Genetic comorbidity of
hypertension and bronchial asthma. Arterial’naya Gipertenziya =
Arterial Hypertension. 2022;28(1):87-95. DOI 10.18705/1607-419X-
2022-28-1-87-95. (in Russian)

Branigan G.L., Soto M., Neumayer L., Rodgers K., Brinton R.D. Association
between hormone-modulating breast cancer therapies and
incidence of neurodegenerative outcomes for women with breast
cancer. JAMA Netw. Open. 2020;3(3):e201541. DOI 10.1001/
jamanetworkopen.2020.1541.

Brown M.S., Goldstein J.L. A receptor-mediated pathway for cholesterol
homeostasis. Science. 1986;232(4746):34-47. DOI 10.1126/
science.3513311.

Brunner H.G., van Driel M.A. From syndrome families to functional
genomics. Nat. Rev. Genet. 2004;5(7):545-551. DOI 10.1038/nrg
1383.

Catalá-López F., Suárez-Pinilla M., Suárez-Pinilla P., Valderas J.M.,
Gómez-Beneyto M., Martinez S., Balanzá-Martínez V., Climent J.,
Valencia A., McGrath J., Crespo-Facorro B., Sanchez-Moreno J.,
Vieta E., Tabarés-Seisdedos R. Inverse and direct cancer comorbidity
in people with central nervous system disorders: a metaanalysis
of cancer incidence in 577,013 participants of 50 observational
studies. Psychother. Psychosom. 2014;83(2):89-105. DOI
10.1159/000356498.

Cohen J., Pertsemlidis A., Kotowski I.K., Graham R., Garcia C.K.,
Hobbs H.H. Low LDL cholesterol in individuals of African descent resulting from frequent nonsense mutations in PCSK9. Nat. Genet.
2005;37(2):161-165. DOI 10.1038/ng1509.

Crespi B.J., Go M.C. Diametrical diseases reflect evolutionary-genetic
tradeoffs: evidence from psychiatry, neurology, rheumatology, oncology
and immunology. Evol. Med. Public. Health. 2015;2015(1):
216-253. DOI 10.1093/emph/eov021.

Davidenkov S.N. Evolutionary Genetic Problems in Neuropathology.
Leningrad: GIDUV Publ., 1947. (in Russian)

Dilman V.M. Aging, Menopause, Cancer. Moscow, 1968. (in Russian)

Diss G., Lehner B. The genetic landscape of a physical interaction.
eLife. 2018;7:e32472. DOI 10.7554/eLife.32472.

Divac A., Nikolic A., Mitic-Milikic M., Nagorni-Obradovic L., Petrovic-
Stanojevic N., Dopudja-Pantic V., Nadaskic R., Savic A., Radojkovic
D. High frequency of the R75Q CFTR variation in patients
with chronic obstructive pulmonary disease. J. Cyst. Fibros.
2004;3(3):189-191. DOI 10.1016/j.jcf.2004.05.049.

Dong G., Feng J., Sun F., Chen J., Zhao X.M. A global overview of
genetically interpretable multimorbidities among common diseases
in the UK Biobank. Genome Med. 2021;13(1):110. DOI 10.1186/
s13073-021-00927-6.

Dzau V.J., Antman E.M., Black H.R., Hayes D.L., Manson J.E., Plutzky
J., Popma J.J., Stevenson W. The cardiovascular disease continuum
validated: clinical evidence of improved patient outcomes. Part I:
Pathophysiology and clinical trial evidence (risk factors through
stable
coronary artery disease). Circulation. 2006;114(25):2850-
2870. DOI 10.1161/CIRCULATIONAHA.106.655688.

Feinstein A.R. The pre-therapeutic classification of co-morbidity in
chronic disease. J. Chronic Dis. 1970;23(7):455-468. DOI 10.1016/
0021-9681(70)90054-8.

Ferreira M.A., Matheson M.C., Tang C.S., Granell R., Ang W., Hui J.,
Kiefer A.K., Duffy D.L., Baltic S., Danoy P., Bui M., Price L.,
Sly P.D., Eriksson N., Madden P.A., Abramson M.J., Holt P.G.,
Heath A.C., Hunter M., Musk B., Robertson C.F., Le Souëf P.,
Montgomery G.W., Henderson A.J., Tung J.Y., Dharmage S.C.,
Brown M.A., James A., Thompson P.J., Pennell C., Martin N.G.,
Evans D.M., Hinds D.A., Hopper J.L., Australian Asthma Genetics
Consortium Collaborators. Genome-wide association analysis
identifies 11 risk variants associated with the asthma with hay fever
phenotype. J. Allergy Clin. Immunol. 2014;133(6):1564-1571. DOI
10.1016/j.jaci.2013.10.030.

Ferreira M.A., Vonk J.M., Baurecht H., Marenholz I., Tian C., Hoffman
J.D., Helmer Q., Tillander A., Ullemar V., van Dongen J., …
Jorgenson E., Lee Y.A., Boomsma D.I., Almqvist C., Karlsson R.,
Koppelman
G.H., Paternoster L. Shared genetic origin of asthma,
hay fever and eczema elucidates allergic disease biology. Nat. Genet.
2017;49(12):1752-1757. DOI 10.1038/ng.3985.

Flannick J., Thorleifsson G., Beer N.L., Jacobs S.B., Grarup N.,
Burtt N.P., Mahajan A., Fuchsberger C., Atzmon G., Benediktsson
R., … Pedersen O., Go-T2D Consortium, T2D-GENES Consortium,
Groop L., Cox D.R., Stefansson K., Altshuler D. Loss-offunction
mutations in SLC30A8 protect against type 2 diabetes. Nat.
Genet. 2014;46(4):357-363. DOI 10.1038/ng.2915.

Forés-Martos J., Boullosa C., Rodrigo-Domínguez D., Sánchez-Valle
J., Suay-García B., Climent J., Falcó A., Valencia A., Puig-Butillé
J.A., Puig S., Tabarés-Seisdedos R. Transcriptomic and genetic
associations between Alzheimer’s disease, Parkinson’s disease, and
cancer. Cancers (Basel). 2021;13(12):2990. DOI 10.3390/cancers
13122990.

Freydin M.B., Ogorodova L.M., Puzyrev V.P. Pathogenetics of Allergic
Diseases. Novosibirsk, 2015. (in Russian)

Gadamer G.-G. On the Circle of Understanding. The Relevance of
Beauty. Moscow, 2010. (in Russian)

Goh K.I., Cusick M.E., Valle D., Childs B., Vidal M., Barabási A.L.
The human disease network. Proc. Natl. Acad. Sci. USA. 2007;
104(21):8685-8690. DOI 10.1073/pnas.0701361104.

Golubovsky M.D. Commentary on the Dialogue on Systematics. Nadezhda
Mandelstam and Lyubishchev. Priroda = Nature. 2006;6:
77-80. (in Russian)

Gomboeva D.E., Bragina E.Y., Nazarenko M.S., Puzyrev V.P. The
inverse comorbidity between oncological diseases and Huntington’s
disease: review of epidemiological and biological evidence.
Russ. J. Genet. 2020;56(3):269-279. DOI 10.1134/S10227954200
30059.

Guglielmotto M., Manassero G., Vasciaveo V., Venezia M., Tabaton
M., Tamagno E. Estrogens inhibit amyloid-β-mediated paired helical
filament-like conformation of tau through antioxidant activity
and miRNA 218 regulation in hTau mice. J. Alzheimers Dis.
2020;77(3):1339-1351. DOI 10.3233/JAD-200707.

Holodkovsky N.A. Lamarckism and Geoffreyism. Priroda = Nature.
1915;4:533-542. (in Russian)

Ibáñez K., Boullosa C., Tabarés-Seisdedos R., Baudot A., Valencia A.
Molecular evidence for the inverse comorbidity between central nervous
system disorders and cancers detected by transcriptomic metaanalyses.
PLoS Genet. 2014;10(2):e1004173. DOI 10.1371/journal.
pgen.1004173.

Jia G., Zhong X., Im H.K., Schoettler N., Pividori M., Hogarth D.K.,
Sperling A.I., White S.R., Naureckas E.T., Lyttle C.S., Terao C., Kamatani
Y., Akiyama M., Matsuda K., Kubo M., Cox N.J., Ober C.,
Rzhetsky A., Solway J. Discerning asthma endotypes through comorbidity
mapping. Nat. Commun. 2022;13(1):6712. DOI 10.1038/
s41467-022-33628-8.

Jones K.L. Hereditary Syndromes According to David Smith. Atlasreference
book. Moscow: Praktika Publ., 2011. (in Russian)

Kathiresan S., Willer C.J., Peloso G.M., Demissie S., Musunuru K.,
Schadt E.E., Kaplan L., Bennett D., Li Y., Tanaka T., … Peltonen L.,
Orho-Melander M., Ordovas J.M., Boehnke M., Abecasis G.R.,
Mohlke
K.L., Cupples L.A. Common variants at 30 loci contribute
to polygenic dyslipidemia. Nat. Genet. 2009;41(1):56-65. DOI
10.1038/ng.291.

Kaznacheev V.P. Modern Aspects of Adaptation. Novosibirsk, 1980.
(in Russian)

Kingston A., Robinson L., Booth H., Knapp M., Jagger C., MODEM
project. Projections of multi-morbidity in the older population in
England to 2035: estimates from the Population Ageing and Care
Simulation (PACSim) model. Age Ageing. 2018;47(3):374-380.
DOI 10.1093/ageing/afx201.

Kolchanov N.A., Ignatieva E.V., Podkolodnaya O.A., Lihoschvai
V.A.
Gene networks. Vavilovskii Zhurnal Genetiki i Selektsii
= Vavilov
Journal of Genetics and Breeding. 2013;17(4-2):833-850. (in Russian)

Krylov A.A. To the problem of compatibility of diseases. Klinicheskaya
Meditsyna = Clinical Medicine. 2000;78(1):56-58. (in Russian)

Lazarevic-Pasti T., Leskovac A., Momic T., Petrovic S., Vasic V. Modulators
of acetylcholinesterase activity: from Alzheimer’s disease to
anti-cancer drugs. Curr. Med. Chem. 2017;24(30):3283-3309. DOI
10.2174/0929867324666170705123509.

Liu X., Li Y.I., Pritchard J.K. Trans effects on gene expression can
drive omnigenic inheritance. Cell. 2019;177(4):1022-1034.e6. DOI
10.1016/j.cell.2019.04.014.

Lupski J.R., Belmont J.W., Boerwinkle E., Gibbs R.A. Clan genomics
and the complex architecture of human disease. Cell. 2011;147(1):
32-43. DOI 10.1016/j.cell.2011.09.008.

Manolio T.A. Bringing genome-wide association findings into clinical
use. Nat. Rev. Genet. 2013;14(8):549-558. DOI 10.1038/nrg
3523.

Mauro A.A., Ghalambor C.K. Trade-offs, pleiotropy, and shared molecular
pathways: a unified view of constraints on adaptation. Integr.
Comp. Biol. 2020;60(2):332-347. DOI 10.1093/icb/icaa056.

McKusick V.A. Some principles of medical genetics. In: Bartalos M. (Ed.)
Genetics in Medical Practice. London: Pitman Medical, 1968;43-54.

McKusick V.A. On lumpers and splitters, or the nosology of genetic
disease. Perspect. Biol. Med. 1969;12(2):298-312. DOI 10.1353/
pbm.1969.0039.

Murmann A.E., Gao Q.Q., Putzbach W.E., Patel M., Bartom E.T.,
Law C.Y., Bridgeman B., Chen S., McMahon K.M., Thaxton C.S.,
Peter M.E. Small interfering RNAs based on huntingtin trinucleotide
repeats are highly toxic to cancer cells. EMBO Rep. 2018;19(3):
e45336. DOI 10.15252/embr.201745336.

Navickas R., Petric V.K., Feigl A.B., Seychell M. Multimorbidity: what
do we know? What should we do? J. Comorb. 2016;6(1):4-11. DOI
10.15256/joc.2016.6.72.

Nazarenko M.S., Slepcov A.A., Puzyrev V.P. “Mendelian code” in the
genetic structure of complex diseases. Genetics.
2022;58(10):1101-
1111. DOI 10.31857/S0016675822100058. (in Russian)

Opitz J.M., Neri G. Historical perspective on developmental concepts
and terminology. Am. J. Med. Genet. A. 2013;161A(11):2711-2725.
DOI 10.1002/ajmg.a.36244.

Paigen K., Eppig J.T. A mouse phenome project. Mamm. Genome.
2000;11(9):715-717. DOI 10.1007/s003350010152.

Palmer C.N., Irvine A.D., Terron-Kwiatkowski A., Zhao Y., Liao H.,
Lee S.P., Goudie D.R., Sandilands A., Campbell L.E., Smith F.J.,
O’Regan G.M., Watson R.M., Cecil J.E., Bale S.J., Compton J.G.,
DiGiovanna J.J., Fleckman P., Lewis-Jones S., Arseculeratne G.,
Sergeant A., Munro C.S., El Houate B., McElreavey K., Halkjaer
L.B., Bisgaard H., Mukhopadhyay S., McLean W.H. Common
loss-of-function variants of the epidermal barrier protein filaggrin
are a major predisposing factor for atopic dermatitis. Nat. Genet.
2006;38(4):441-446. DOI 10.1038/ng1767.

Pepe P., Vatrano S., Cannarella R., Calogero A.E., Marchese G.,
Ravo M., Fraggetta F., Pepe L., Pennisi M., Romano C., Ferri R.,
Salemi M. A study of gene expression by RNA-seq in patients with
prostate cancer and in patients with Parkinson disease: an example
of inverse comorbidity. Mol. Biol. Rep. 2021;48(11):7627-7631.
DOI 10.1007/s11033-021-06723-0.

Pfaundler M., Seht L.V. Über Syntropie von Krankheitszuständen.
Z. Kinder-Heilk. 1921;30:100-120. DOI 10.1007/BF02222706.

Piro R.M. Network medicine: linking disorders. Hum. Genet. 2012;
131(12):1811-1820. DOI 10.1007/s00439-012-1206-y.

Puzyrev V.P. Genetic bases of human comorbidity. Russ. J. Genet.
2015;51(4):408-417. DOI 10.1134/S1022795415040092.

Puzyrev V.P., Makeeva O.A., Freidin M.B. Syntropy, genetic testing
and personalized medicine. Per. Med. 2010;7(4):399-405. DOI
10.2217/pme.10.35.

Puzyryov V.P. Liberties of genome and medical pathogenetics. Byulleten
Sibirskoy Meditsiny = Bulletin of Siberian Medicine. 2002;1(2):
16-29. DOI 10.20538/1682-0363-2002-2-16-29. (in Russian)

Quick C.R., Conway K.P., Swendsen J., Stapp E.K., Cui L., Merikangas
K.R. Comorbidity and coaggregation of major depressive disorder
and bipolar disorder and cannabis use disorder in a controlled
family study. JAMA Psychiatry. 2022;79(7):727-735. DOI 10.1001/
jamapsychiatry.2022.1338.

Reaven G.M. Banting lecture 1988. Role of insulin resistance in human
disease. Diabetes. 1988;37(12):1595-1607. DOI 10.2337/diab.37.
12.1595.

Reynolds R.J., Irvin M.R., Bridges S.L., Kim H., Merriman T.R., Arnett
D.K., Singh J.A., Sumpter N.A., Lupi A.S., Vazquez A.I. Genetic
correlations between traits associated with hyperuricemia, gout,
and comorbidities. Eur. J. Hum. Genet. 2021;29(9):1438-1445. DOI
10.1038/s41431-021-00830-z.

Rijken M., Hujala A., van Ginneken E., Melchiorre M.G., Groenewegen
P., Schellevis F. Managing multimorbidity: profiles of integrated
care approaches targeting people with multiple chronic conditions
in Europe. Health Policy. 2018;122(1):44-52. DOI 10.1016/j.health
pol.2017.10.002.

Rubio-Perez C., Guney E., Aguilar D., Piñero J., Garcia-Garcia J., Iadarola
B., Sanz F., Fernandez-Fuentes N., Furlong L.I., Oliva B. Genetic
and functional characterization of disease associations explains
comorbidity. Sci. Rep. 2017;7(1):6207. DOI 10.1038/s41598-017-
04939-4.

Ryu I., Ryu M.J., Han J., Kim S.J., Lee M.J., Ju X., Yoo B.H., Lee Y.L.,
Jang Y., Song I.C., Chung W., Oh E., Heo J.Y., Kweon G.R. L-Deprenyl
exerts cytotoxicity towards acute myeloid leukemia through inhibition
of mitochondrial respiration. Oncol. Rep. 2018;40(6):3869-
3878. DOI 10.3892/or.2018.6753.

Rzhetsky A., Wajngurt D., Park N., Zheng T. Probing genetic overlap
among complex human phenotypes. Proc. Natl. Acad. Sci. USA.
2007;104(28):11694-11699. DOI 10.1073/pnas.0704820104.

Sandilands A., Terron-Kwiatkowski A., Hull P.R., O’Regan G.M., Clayton
T.H., Watson R.M., Carrick T., Evans A.T., Liao H., Zhao Y.,
Campbell L.E., Schmuth M., Gruber R., Janecke A.R., Elias P.M.,
van Steensel M.A., Nagtzaam I., van Geel M., Steijlen P.M., Munro
C.S., Bradley D.G., Palmer C.N., Smith F.J., McLean W.H., Irvine
A.D. Comprehensive analysis of the gene encoding filaggrin uncovers
prevalent and rare mutations in ichthyosis vulgaris and atopic
eczema. Nat. Genet. 2007;39(5):650-654. DOI 10.1038/ng2020.

Serebrovskiy A.S. Some Problems of Organic Evolution. Moscow,
1973. (in Russian)

Shadrina A.S., Sharapov S.Z., Shashkova T.I., Tsepilov Y.A. Varicose
veins of lower extremities: insights from the first large-scale genetic
study. PLoS Genet. 2019;15(4):e1008110. DOI 10.1371/journal.
pgen.1008110.

Shnayder N.A., Novitsky M.A., Neznanov N.G., Limankin O.V., Asadullin
A.R., Petrov A.V., Dmitrenko D.V., Narodova E.A., Popenko
N.V., Nasyrova R.F. Genetic predisposition to schizophrenia and
depressive disorder comorbidity. Genes (Basel). 2022;13(3):457.
DOI 10.3390/genes13030457.

Sidransky E., Nalls M.A., Aasly J.O., Aharon-Peretz J., Annesi G.,
Barbosa E.R., Bar-Shira A., Berg D., Bras J., Brice A., … Tsuji S.,
Wittstock M., Wolfsberg T.G., Wu Y.R., Zabetian C.P., Zhao Y.,
Ziegler S.G. Multicenter analysis of glucocerebrosidase
mutations
in Parkinson’s disease. N. Engl. J. Med. 2009; 361(17):1651-1661.
DOI 10.1056/NEJMoa0901281.

Spataro N., Rodríguez J.A., Navarro A., Bosch E. Properties of human
disease genes and the role of genes linked to Mendelian disorders in
complex disease aetiology. Hum. Mol. Genet. 2017;26(3):489-500.
DOI 10.1093/hmg/ddw405.

Stein O., Stein Y. Smooth muscle cells and atherosclerosis. Curr. Opin.
Lipidol. 1995;6(5):269-274. DOI 10.1097/00041433-199510000-
00005.

Stephens Z.D., Lee S.Y., Faghri F., Campbell R.H., Zhai C., Efron M.J.,
Iyer R., Schatz M.C., Sinha S., Robinson G.E. Big data: astronomical
or genomical? PLoS Biol. 2015;13(7):e1002195. DOI 10.1371/
journal.pbio.1002195.

Vertkin A.L. Comorbid Patient. Moscow, 2015. (in Russian)

Vertkin A.L., Rumyantsev M.A., Skotnikov A.S. Comorbidity. Klinicheskaya
Meditsyna = Clinical Medicine. 2012;90(10):4-11. (in
Russian)

Vilenkin A. Many Worlds in One. The Search for Other Universes.
Moscow: Astrel Publ., 2010. (in Russian)

Vyatkin V.B. About application of the term “syntropy” in scientific research.
Nauchnoye Obozreniye. Referativnyy Zhurnal = Scientific
Review. Abstract Journal. 2016;3:81-84. (in Russian)

Wagner G.P., Kenney-Hunt J.P., Pavlicev M., Peck J.R., Waxman D.,
Cheverud J.M. Pleiotropic scaling of gene effects and the ‘cost of
complexity’. Nature. 2008;452(7186):470-472. DOI 10.1038/nature
06756.

Wang M., Tang S., Yang X., Xie X., Luo Y., He S., Li X., Feng X.
Identification of key genes and pathways in chronic rhinosinusitis with nasal polyps and asthma comorbidity using bioinformatics approaches.
Front. Immunol. 2022;13:941547. DOI 10.3389/fimmu.
2022.941547.

Weiss F.U., Simon P., Bogdanova N., Mayerle J., Dworniczak B.,
Horst J., Lerch M.M. Complete cystic fibrosis transmembrane
conductance regulator gene sequencing in patients with idiopathic
chronic pancreatitis and controls. Gut. 2005;54(10):1456-1460. DOI
10.1136/gut.2005.064808.

Werner H., Sarfstein R., Nagaraj K., Laron Z. Laron syndrome research
paves the way for new insights in oncological investigation. Cells.
2020;9(11):2446. DOI 10.3390/cells9112446.

Woese C.R., Goldenfeld N. How the microbial world saved evolution
from the scylla of molecular biology and the charybdis of the modern
synthesis. Microbiol. Mol. Biol. Rev. 2009;73(1):14-21. DOI
10.1128/MMBR.00002-09.

Wray N.R., Goddard M.E., Visscher P.M. Prediction of individual genetic
risk of complex disease. Curr. Opin. Genet. Dev. 2008;18(3):
257-263. DOI 10.1016/j.gde.2008.07.006.

Zhao R., Choi B.Y., Lee M.H., Bode A.M., Dong Z. Implications of
genetic and epigenetic alterations of CDKN2A (p16INK4a) in cancer.
EBioMedicine. 2016;8:30-39. DOI 10.1016/j.ebiom.2016.04.017.

Zolotareva O., Saik O.V., Königs C., Bragina E.Y., Goncharova I.A.,
Freidin M.B., Dosenko V.E., Ivanisenko V.A., Hofestädt R. Comorbidity
of asthma and hypertension may be mediated by shared genetic
dysregulation and drug side effects. Sci. Rep. 2019;9(1):16302.
DOI 10.1038/s41598-019-52762-w.

